# Alexithymia increases effects of ego-depletion

**DOI:** 10.3389/fpsyt.2022.970244

**Published:** 2022-10-19

**Authors:** Konrad Schnabel, Olga Pollatos

**Affiliations:** ^1^International Psychoanalytic University Berlin, Berlin, Germany; ^2^Clinical and Health Psychology, Institute of Psychology and Education, Ulm University, Ulm, Germany

**Keywords:** alexithymia, ego-depletion, cognitive control, pain tolerance, Stroop interferences

## Abstract

Alexithymia is associated with deficiencies to identify, describe and express emotions, paucity of fantasies and an externally oriented cognitive style. The current studies provide evidence that alexithymia is also related to self-regulation processes and exacerbates effects of ego-depletion, a state where self-regulation resources are reduced due to previous acts of self-regulation. In Study 1, ego-depletion effects of a handgrip task on pain tolerance were increased by alexithymia. In Study 2, an emotion suppression task showed stronger effects of ego-depletion on a Stroop task in participants high rather than low in alexithymia, but only after suppression of emotions induced by negative and not positive pictures. The results imply that alexithymia increases susceptibility to ego-depletion effects, that consumption of self-regulatory resources has stronger consequences for people high in alexithymia and that they should experience special support in ways to replenish self-regulation resources.

## Introduction

Alexithymia is a multidimensional construct consisting of cognitive and affective characteristics: Deficiency to identify, describe and express emotions, paucity of fantasies, and an externally oriented cognitive style (1). Within clinical and non-clinical populations, alexithymia is viewed as a continuous personality trait which can be assessed by the Toronto Alexithymia Scale (TAS-20; 1). In several behavioral studies, alexithymia is thought to reflect deficits in the cognitive processing and regulation of emotions. For instance, participants high in alexithymia showed slower responses in the attentional blink paradigm after processing facial expressions of fear and pain as compared to participants low in alexithymia (2). Similarly, participants with high alexithymia scores showed poor emotion regulation strategies and impeded processing of emotional stimuli (3). Alexithymia related deficits in emotional processing are also viewed as a source of emotional over-responding (e.g., impulsive or aggressive behaviors) due to limited cognitive and affective resources that are essential to successfully cope with stressful experiences (4). The current studies explored how alexithymia is related to self-regulation on a more general level. Self-regulation is a highly adaptive, distinctively human trait that enables people to pursue personal goals and to alter their responses [see e.g., (5)]. Several studies demonstrated that self-regulation consumes a limited resource, thereby creating a temporary state of ego depletion [e.g., (6)]. Ego-depletion represents a state of reduced physiological, cognitive, or emotional resources that increases the likelihood of subsequent self-regulation failures. Ego-depletion effects are tested using a sequential task paradigm in which participants complete at least two tasks that require self-regulation. Depletion effects are evident, if self-regulation efforts that are preceded by self-regulation requests are less successful than without previous acts of self-control. Ego-depletion can be defined as a state of short-term self-regulatory deficits that are caused by previous affective, cognitive, or physical self-regulation efforts. Meta-analyses revealed mixed results with respect to the replicability of ego-depletion effects. An earlier meta-analysis showed medium-to-large ego-depletion effects across a variety of domains such as cognitive and physical tasks, risk taking, and health related behavior (7). Importantly, depletion effects were unaffected by whether the depleting and the dependent tasks belonged to identical or different domains. More recent meta-analytical approaches failed to replicate ego-depletion effects in sequential task paradigms using preregistered protocols across multiple labs (8, 9). Depletion effects from sequential tasks may be attributed to motivational or attentional changes rather than depleted resources (10) and self-control efforts that buffer against depletion effects may operate automatically and without conscious awareness (11). Limited and inconsistent empirical evidence in support of ego-depletion effects [cf. (12)] highlight the relevance of searching for possible moderators like alexithymia that may influence the size of ego-depletion effects. As stated previously, there is growing empirical evidence suggesting that alexithymia is associated with deficits in emotional processing and emotion regulation abilities. Therefore, ego-depletion effects could be modulated by alexithymia. A recent study by Aydogmus and Hamilton (13) showed that emotion-processing deficits in a sample of patients suffering from medically-unexplained physical symptoms were associated with more pronounced ego-depletion effects using an emotional viewing paradigm. Also the bi-directional effects between exercise and self-control were extensively investigated [see review by Boat and Cooper (14)]. Pain perception is known to be altered after cognitive-demanding tasks (5, 15). It can be concluded that tasks referring to somatic sensations such as pain are useful to investigate the possible relationship between emotion regulation, self-control and ego-depletion and that tasks using physical effort might be a promising avenue to induce ego-depletion effects when taking alexithymia into account.

However, the question whether ego-depletion effects on subsequent pain perception are modulated by alexithymia trait still needs to be addressed. It can be hypothesized that effects of ego depletion on pain measures and hereby especially on those related to affective components such as pain tolerance are positively associated with alexithymia. This question is of high clinical relevance as there is numerous empirical evidence reporting high alexithymia in samples suffering from chronic pain (due to various reasons) or somatoform pain disorders [see e.g., (16)].

The current studies aimed to explore whether cognitive and emotional deficits related to alexithymia also exacerbate effects of ego-depletion that are characterized by a state of reduced cognitive self-control resources. It is known that alexithymia hampers effective regulation of emotion and interacts with the perception of emotional stimuli (17, 18). It has been suggested that these deficits may in turn result in a state of negative affect that fosters a hypervigilance toward somatic sensations (19). A study by Inzlicht and Gutsell (20) showed that participants who had to suppress their emotions while watching an emotionally demanding movie performed worse in a subsequent color naming Stroop task. Previous studies showed that alexithymia is related to cognitive deficits in emotional processing and especially to impaired emotional attention [e.g., (21)]. Therefore, it can be hypothesized that ego-depletion effects following emotion suppression are more pronounced in participants with high rather than low alexithymia scores. Our studies used two different approaches to induce ego-depletion, i.e., a physical effort task (handgrip task prior to pain perception) and an emotion suppression task, and two different ways to assess ego-depletion effects, i.e., pain perception measures such as pain threshold and pain tolerance as well as the Stroop task. These studies are the first that explored how alexithymia modulates effects of ego-depletion.

### Study 1

Study 1 explored whether ego-depletion effects of a physical effort task (handgrip task) on subsequently examined pain measures and thereby especially to experimentally assessed pain tolerance are positively related to higher alexithymia as assessed by questionnaire.

## Methods

### Participants

Participants were screened concerning handedness (22) and their health status using an anamnestic questionnaire. Participants were only included if they were right-handed (main ratio therefore was to use an identical set-up in the physical effort task) and did not have a history of any axis 1 disorder, in particular anxiety disorders or depression according to the Diagnostic and Statistical Manual of Mental Disorders (23). Drug use (except of contraceptives) and high levels or regular frequency of everyday pain were additional exclusion criteria. All participants gave their written informed consent. They received an amount of 10€ for their participation. A total of 115 participants (57 males) took part, their mean age was 26.10 (*SD* = 5.80). Experiments were conducted in accordance with the Declaration of Helsinki with the approval of the local ethics committee. The sample size allowed to discover effects in the regression analysis (*N* = 100 sample size with small to medium estimated effect size 0.10, α = 0.05 and β = 0.80; G^*^Power version 3.1.9.7).

### Procedure

Upon arrival at the laboratory, participants first filled in a demographic questionnaire, a questionnaire exploring the individual history of pain experience and the TAS-20 and were then randomly assigned to either start with the high effort condition or the low effort condition using a 3 min isometric exercise task. First individual gripping strength was determined using the BIOPAC SS25 isometric dynamometer always using the left hand. This individually assessed gripping strength was used as 100% reference score for the two subsequent experimental manipulations. The assessment of the individual gripping strength took about 3 min, and there was a break of about 5 min after the assessment before the main experiment started. The order of both conditions used in the main experiment (handgrip with low effort vs. high effort) was randomly assigned. In the low effort condition a light isometric exercise was performed using a handgrip task at about 10% of each subject's maximal voluntary contraction which had to be sustained for 3 min with the left hand. This task was the control condition with no expected effect on physical activity and on self-regulatory processes. The high effort condition corresponded to about 50% of each subject's maximal voluntary contraction to be sustained for 3 min [see e.g., (24)] and was expected to deplete participants.

Immediately after each handgrip task, pressure pain thresholds and tolerance were measured with a pressure algometer (FDN200, Wagner Instruments, USA) that exerts forces up to 20 kg/cm^2^ (corresponding to ~2,000 kPa). This validated method has a high inter-rater reliability in the rate of force application (25). Before testing, all experimenters were made familiar with the algometer in practice sessions. The handheld algometer had a 1 cm^2^ round rubber application surface, which was placed over the thenar eminence [see e.g., (26)] of the right hand. Pressure pain threshold (PPT) was determined with three series of ascending stimulus intensities, each applied as a slowly increasing ramp of 50 kPa/s (~0.5 kg/cm 2 s). This procedure leads to high reliability of the algometer assessment and is in accordance to former studies (24). Each trial was stopped when the participant experienced the pressure applied by the algometer as “painful.” Pressure pain tolerance level (PTOL) was then assessed with the trial when participants experienced the pressure as “unbearable.” After this first pain assessment a 15 min break was introduced before the second condition started. The whole experiment lasted about 30 min.

### Data analyses

Depletion effect on ain measures were analyzed using repeated measures analyses with *Condition* (ego-depletion vs. control condition) as a within subjects factor and order of the conditions (first control, second ego-depletion; and vice versa) as covariate. In case of a significant main effect of condition a multivariate regression analysis was calculated using the z-transformed delta score between both conditions as criterion and the z-transformed TAS total scores as well as the order of the conditions as predictors (forward selection). In the Results section, uncorrected F-values are reported together with the Greenhouse-Geiser epsilon values and corrected degrees of freedom.

## Results

### TAS-20

The 20-item Toronto Alexithymia Scale (TAS-20; 1) measures three dimensions of the alexithymia construct: difficulty identifying emotions (DIF; e.g., “I am often confused about what emotion I am feeling”); difficulty describing emotions (DDF; e.g., “It is difficult for me to find the right words for my feelings”); and externally oriented thinking (EOT; e.g., “I prefer talking to people about their daily activities rather than their feelings”). A total score is calculated summing up the three subscores. Numerous studies attest to the good internal consistency of the TAS 20. In the present study, internal consistency of the TAS 20 was Cronbach's α = 0.83.The mean total alexithymia score was *M* = 39.17 (*SD* = 9.06). While TAS-20 scores between 52 and 60 are usually described as light alexithymia and scores above 60 as high alexithymia (1), two participants showed high and five participants showed light alexithymia according to this classification.

### Pain threshold

Concerning pressure pain threshold, no significant main effect of *Condition* (mean threshold control condition *M* = 3.06; mean threshold after ego depletion *M* = 3.02; *F*( 1.113) = 0.00; *p* = 0.99.) occurred. The covariate *Order* was not significant (*F* (1.113) = 0.03, *p* = 0.77). Furthermore, no significant interaction effects between *Order X Condition* (*F* (1.113) = 0.03, *p* = 0.86) occurred.

### Pain tolerance

Concerning pressure pain tolerance, the ANOVA revealed a significant main effect of *Condition* (*F* (1.113) = 4.60; *p* = 0.34; η^2^ = 0.04; ε = 0.57): Pressure pain tolerance was significantly decreased after ego depletion as compared to the control condition, *M* = 6.13 versus *M* = 5.30. No main effect of *Order* (*F* (1.113) = 0.23; *p* = 0.63) and no interaction effect *Order x Condition* (*F* (1.113) = 0.01; *p* = 0.93) were observed. In the subsequent regression analysis a significant effect of alexithymia was revealed (T = −2.87, β = −0.26, *p* = 0.005; *F* (1.113) = 8.25, *p*= *0.005, R* = 0.26, *R*^2^ = 0.07). Higher alexithymia was associated with a more pronounced drop in pain tolerance (β = −0.26).

## Discussion

Study 1 showed that alexithymia modulated the after-effect of cognitive control on pain. Reduction of pressure pain tolerance after ego-depletion was more pronounced for participants with higher alexithymia scores. To our knowledge this is the first study that could demonstrate that alexithymia interacts with the after-effect of ego-depletion on pain measures, highlighting that alexithymia is associated not only with self-regulatory capacities, but also modulates the after-effects of depleting these self-regulatory resources. In accordance to other studies [e.g., (5)] we could also show that the resource depletion effect held only for pain tolerance while the sensory-discriminative component of pain was not significantly modulated by after-effects of cognitive control. In relation to our results, it can be hypothesized that alexithymia might either be associated with greater effort in self-regulatory processes as induced by a physical effort task in this study or by a greater difficulty to fill up the storage used for self-control. Therefore, the depletion of the resource for self-control causes more impairment in alexithymia in subsequent self-regulatory processes.

Potential limitations of the current study refer to the fact that additional pain modulation might be caused in result of the isometric exercise task used to induce ego-depletion. Several experimental studies have examined the effects of acute exercise on pain modulation in subjects with or without pain, commonly called exercise-induced hypoalgesia [see e.g., (27)]. Results showed that isometric exercise also produces hypoalgesia at both low and high intensity [e.g., (28)]. A possible limitation derives from the dynamometer used in this study. This procedure might be influenced by cognitive control in a stronger fashion as, for instance, automatically applied electric stimulation. Another important shortcoming of the present study is that we did not assess pain threshold and pain tolerance in a baseline condition without any ego-depletion task. Even though we assume that the light isometric exercise does not differ significantly with respect to pain measures, we cannot rule out that any kind of exercise does interact with alexithymia. A limitation of this study refers also to the fact that only few participants exhibited high or very high alexithymia total scores suggesting that most of the investigated participants lie within high functionality. It is therefore an open point whether the same effects are to be observed in a highly affected e.g., clinical population. Future research should address these open questions, e.g., by assessing pain measures on several days with and without any manipulation of self-regulatory capacity or by including also highly affected participants with respect to their TAS scores.

### Study 2

Study 2 explored whether ego-depletion effects of emotion suppression on a Stroop task were influenced by the alexithymia scores of participants. Extending the results obtained in Study 1 using physical effort to induce ego-depletion, we decided to use an emotional suppression task, because alexithymia is linked to problems in both emotional processing as well as emotion regulation. We therefore expected larger ego-depletion effects after an emotional suppression task for participants with high rather than low alexithymia scores.

## Methods

### Participants

The sample consisted of 82 (59 females) psychology students with a mean age of 22.89 (*SD* = 4.55) years. Participants received course credit for their participation. The sample size allowed to discover mean differences of *d* = 0.55 and correlations of *r* = 0.27 with α = 0.05 and β = 0.80.

### Overview of procedure and design

Data were collected individually at the lab. Participants were informed that the study explored the effects of emotion perception and that it contained questionnaire measures and sorting tasks. Participants first completed the TAS-20 which was followed by the first emotion suppression task using 10 negative pictures followed by the first Stroop task. Finally, participants completed the second emotion suppression task using 10 positive pictures followed by the second Stroop task.

### Emotion suppression tasks

The emotion suppression task instructed participants to watch a series of 10 pictures while intentionally suppressing all internal reactions and external signs of their feelings such as facial expressions or gestures. Participants completed two emotion suppression tasks with the first presenting negative pictures and the second presenting positive pictures. Pictures were presented for 10 seconds and were taken from the International Affective Picture System [IAPS; (29)].

### Stroop tasks

We used a German version of the standard color word Stroop task from millisecond.com. Stimuli consisted of the words “red”, “green”, “blue”, and “black” presented in either the color they referred to (congruent trials) or in one of the other three colors (incongruent trials). Control trails consisted of a rectangle displayed in one of the four colors. Subjects responded to each trial by pressing the “d”, “f”, “j”, or “k” key, if the stimulus was presented in red, green, blue, or black color, respectively, while they had to ignore the meaning of the color words. Stimulus presentation started after an inter-trial interval of 200 milliseconds. After erroneous responses a red “X” was displayed for 400 milliseconds. The Stroop task consisted of a total of 84 trials and congruent, incongruent, and control trials each contributed one third of the trials. Order of trials was randomized. Performance in the Stroop task was calculated as the difference in mean response latencies between the congruent and the incongruent trials with high scores indicating slower responses in the incongruent rather than the congruent trials. The Stroop task was completed twice. The first Stroop immediately followed the emotion suppression task displaying negative pictures. The second Stroop immediately followed the emotion suppression task displaying positive pictures.

## Results

### TAS-20 and stroop

Internal consistency of the TAS was Cronbach's α = 0.85. Internal consistencies for the Stroop were estimated as split-half reliabilities and were Cronbach's α = 0.36 and.47 for the first and second Stroop task, respectively. Due to the low reliabilities of the Stroop scores, we did not only use correlational analyses to explore the relationship between alexithymia and the Stroop, but we also compared mean differences between groups of participants with high vs. low alexithymia scores. The mean TAS-20 score was *M* = 37.84 (*SD* = 10.08). While TAS-20 scores between 52 and 60 are usually described as light alexithymia and scores above 60 as high alexithymia (1), two participants showed high and seven participants showed light alexithymia according to this classification. However, alexithymia also represents a continuous variable (1). Therefore, we compared high vs. low alexithymia scores and also report correlations between alexithymia and ego-depletion effects.

Using a median split procedure, 41 participants (eight males) were classified as low in alexithymia (*M* = 29.92, *SD* = 3.57, range 24–36) and 41 participants (10 males) as high in alexithymia (*M* = 45.76, *SD* = 8.03, range 37–77). Both groups were comparable concerning their sex distribution and did not show significant age differences (high vs. low TAS-20, *M* = 22.46, *SD* = 4.12 vs. *M* = 23.40, *SD* = 5.00, *t*(79) = −0.92, *p* =.36, *d* = −0.21). Larger Stroop effects in the first Stroop task were evident for participants high in alexithymia (*M* = 261.65, *SD* = 165.29) than for participants low in alexithymia (*M* = 167.86, *SD* = 198.44), *t* (80) = 2.33, *p* = 0.02, *d* = 0.52. In contrast, both groups did not differ significantly in the second Stroop task (high vs/ low TAS-20, *M* = 188.83, *SD* = 219.36 vs. *M* = 171.13, *SD* = 117.98, *t* (80) = 0.45, *p* = 0.65, *d* = 0.10). An association between the TAS-20 and the first Stroop task was also mirrored by the positive correlation between these measures, *r* = 0.23, *p* = 0.04. In contrast, the TAS-20 did not correlate with the second Stroop task, *r* = 0.05, *p* = 0.63.

## Discussion

The results revealed stronger ego-depletion effects as indicated by Stroop interferences for participants with high rather than low TAS-20 scores. However, individual differences in alexithymia modulated ego-depletion effects only in the first Stroop task after the suppression of negative affect whereas no significant effects of alexithymia were visible in the second Stroop task after the suppression of positive affect. Because we wanted participants to always end the experiment after the presentation of positive pictures, our design confounded position effects and effects of affective valence. As a consequence, the current results could not elucidate whether alexithymia would modulate ego-depletion effects also after the suppression of positive affect, if position and learning effects on the Stroop task were controlled for. Practice effects might have decreased possible ego-depletion effects on the second Stroop task. Evidence that negative rather than positive affect is linked to ego-depletion stems from the meta-analysis by Hagger et al. (7) showing significant post-depletion increases in negative affect and no effect on positive affect. The processing of negative emotions may add on the demanding and aversive nature of the depleting task and therefore be a source of stronger ego-depletion effects as compared to the processing of positive emotions. In accordance with Study 1, participants with high TAS-20 scores showed stronger ego-depletion effects than participants with low TAS-20 scores. The findings of both studies corroborate the view that alexithymia impairs the processing of emotions and can therefore also have negative effects on the performance in cognitive tasks. As a limitation and differently from Study 1, Study 2 did not exclude participants with depression or anxiety disorders. Consequently, the results can not elucidate to what extent the effects can be completely attributed to differences in alexithymia or might have been influenced by other psychopathological traits. Participants with higher levels of negative affect, might have had more difficulties to suppress responses to negative stimuli. Study 2 also did not include a manipulation check that showed that participants were in fact following the instructions and inhibited their emotions. Finally, Study 2 lacked a baseline Stroop task showing that emotion inhibition produced ego-depletion effects in a repeated measures design and used comparisons between participants with high vs. low alexithymia scores instead.

## General discussion

The current studies provided evidence that interindividual differences in alexithymia play a significant role when dealing with ego depletion after acts of self-regulation. In Study 1, higher alexithymia scores were associated with lower pain tolerance after ego-depletion induced by physical effort, i.e., ahandgrip task. Stronger ego-depletion effects for participants with high alexithymia scores were replicated in Study 2 using a different ego-depletion paradigm and a different dependent variable. Participants were instructed to suppress all affective reactions while watching sad pictures and their cognitive functioning as indicated by interferences in a color naming Stroop task was measured. Again, participants with high rather than low alexithymia scores showed stronger Stroop interferences. This effect did not emerge, when participants were instructed to suppress their emotions while watching positive pictures. Whether this was an effect of position (the negative picture task came always first) or an emotion-specific effect could not be elucidated with the present design. Future studies should investigate to what extent alexithymia predominantly refers to difficulties to experience and describe negative emotions or whether alexithymia also has an impact on processing positive emotions. Because alexithymia is closely related to negative and positive mood [e.g., (30)] future studies should also explore to what extent the current results may be influenced by mood differences. Importantly, alexithymia was not only related to effects of ego-depletion when the ego-depletion task was clearly emotional (i.e., emotion suppression in Study 2), but also when the ego-depletion task was non-emotional (i.e., physical handgrip task in Study 1). A relevant shortcoming refers to the fact that the amount of depressive symptomatology in a subclinical range was not assessed by e.g., common questionnaires and therefore we cannot rule out that depression might interact with the observed results in both studies.

Our results have implications for interventions dealing with the replenishment of self-regulation resources after ego-depletion. Previous research indicates that positive affect and rest help to recharge the batteries feeding self-regulation processes and may therefore extinguish negative after-effects of ego-depletion. The current studies imply that problems to identify and describe emotions exacerbate demands resulting from self-regulation processes and accelerate the depletion of their resources. Interventions that have the potential to counteract the higher vulnerability for ego-depletion resulting from alexithymia may point in this direction. Individuals high in alexithymia may generally profit from trainings that help them to more accurately perceive own emotions and to become more self-secure with respect to their own emotional processes. This may override negative effects stemming from a lack of emotional clarity and, as a consequence, preserve self-regulation resources. Because alexithymic individuals show patterns of avoiding emotional experiences, the treatment of alexithymia is challenging [cf. (31)]. Treatment targets are to promote mental representations of emotions, to develop the identification of feelings and to offer opportunities for their regulation. Treatment should support the transition from implicit and diffuse to more explicit and self-reflected processing of emotions. Treatment should also acknowledge that alexithymic individuals may be particularly challenged by self-regulatory efforts and that they should be given special opportunities to relax and to manage their individual self-regulation potentials. Because ego-depletion effects provide a serious threat to successful self-regulation, future research should make stronger efforts in order to develop means and ways to buffer against ego-depletion and to protect self-regulation resources.

## Data availability statement

The original contributions presented in the study are publicly available. The data and syntax files can be found here: https://osf.io/se7du.

## Ethics statement

The studies involving human participants were reviewed and approved by University of Potsdam, Psychology Department. The patients/participants provided their written informed consent to participate in this study.

## Author contributions

All authors listed have made a substantial, direct, and intellectual contribution to the work and approved it for publication.

## Conflict of interest

The authors declare that the research was conducted in the absence of any commercial or financial relationships that could be construed as a potential conflict of interest.

## Publisher's note

All claims expressed in this article are solely those of the authors and do not necessarily represent those of their affiliated organizations, or those of the publisher, the editors and the reviewers. Any product that may be evaluated in this article, or claim that may be made by its manufacturer, is not guaranteed or endorsed by the publisher.
